# Parameters Influencing Fracture Strength in Implant-Supported Zirconia Restorations: A Scoping Review

**DOI:** 10.3390/jfb17080391

**Published:** 2026-08-08

**Authors:** Sven Gojsovic, Vladimir Prpic, Amir Catic

**Affiliations:** 1 Department of Fixed Prosthodontics, School of Dental Medicine, University of Zagreb, Gunduliceva 5, 10000 Zagreb, Croatia; sgojsovic@sfzg.unizg.hr; 2 Department of Fixed Prosthodontics, School of Dental Medicine, University of Zagreb, Clinical Hospital Centre Zagreb, Gunduliceva 5, 10000 Zagreb, Croatia; catic@sfzg.unizg.hr

**Keywords:** zirconia, fracture strength, fracture load, implant-supported restoration, prosthodontics, generative design

## Abstract

Mechanical properties are considered key determinants of the long-term success of implant-supported zirconia restorations. However, limited data are available regarding the parameters that influence the fracture strength of implant-supported zirconia restorations. Generative design is well suited for the optimization in implant prosthodontics, as it allows the definition of a constrained design space and the application of optimization algorithms to evaluate numerous design iterations based on predefined biomechanical objectives. Prior to the implementation of generative design, it is essential to identify and define the parameters that influence the optimization of structures such as hybrid implant abutments. The parameters identified in this review will be incorporated into the parametric model and subsequently applied within the generative design workflow to facilitate the customization of hybrid implant abutments. A comprehensive literature search was conducted in the PubMed and Scopus databases to identify relevant studies published between 1 December 2025 and 1 May 2026. In vitro studies investigating fracture strength of implant-supported zirconia restorations were considered eligible for inclusion. A total of 12 studies met the eligibility criteria. Their findings indicated that implant-supported zirconia restorations generally withstood fracture loads exceeding the maximum masticatory forces reported in the oral environment, although the recorded values varied according to restoration design, abutment type, surface modifications, material thickness, retention type, material selection, luting agents, and interface characteristics.

## 1. Introduction

Implant prosthodontic therapy has become a widely accepted treatment modality for the replacement of missing teeth, enabling the restoration of masticatory function while simultaneously providing favorable esthetic outcomes [1]. Recent developments in biomaterials and manufacturing technologies have considerably expanded the clinical indications for metal-free restorations in implant prosthodontic dentistry [2,3,4]. In particular, all-ceramic restorations have emerged as widely used treatment options because of their superior esthetic integration, favorable biological response, and improved optical properties [2]. These properties are particularly evident when compared with traditional metal-ceramic restorations. The widespread adoption of Computer-Aided Design/Computer-Aided Manufacturing (CAD/CAM) technology has been central to this transition, enabling the precise digital fabrication of ceramic restorations with high reproducibility and minimal manual error [3,4]. Among all-ceramic restorations, zirconia has distinguished itself due to its excellent mechanical properties. Its dense and highly cohesive microstructure provides high resistance to fracture, making it one of the strongest materials currently used for dental restorations [5].

Among currently available restorative materials, yttria-stabilized tetragonal zirconia polycrystal (Y-TZP) stands out for its exceptional mechanical strength, favorable biocompatibility, and high resistance to corrosion. Yttria-stabilized tetragonal zirconia polycrystal exists in three crystallographic phases depending on temperature: the monoclinic phase at room temperature, the tetragonal phase above approximately 1170 °C, and the cubic phase above approximately 2370 °C. Among these, the tetragonal phase exhibits the highest mechanical strength and durability, making it the most desirable for biomedical applications. To retain the tetragonal structure at room temperature, yttria (Y_2_O_3_) is incorporated into the zirconia powder as a stabilizing oxide, thereby preventing spontaneous transformation to the monoclinic phase and enhancing the material’s mechanical stability [6]. Consequently, the widespread adoption of zirconia in prosthodontics can be largely attributed to its favorable mechanical properties.

Zirconia exists in several generations, each offering a different balance between mechanical strength and optical translucency. The first generation of zirconia consisted of zirconia stabilized with 3 mol% yttria and containing approximately 0.25 wt% alumina, commonly referred to as 3Y-zirconia. This material was characterized by high flexural strength and fracture toughness; however, its optical properties were limited due to low translucency. Despite its favorable mechanical performance and cost-effectiveness, further modifications were required to improve translucency and enhance its esthetic acceptance in dental restorative applications. The second generation of zirconia contains more than 70% tetragonal phase and less than 30% cubic phase, depending on the sintering temperature, and is also commonly classified as 3Y-zirconia. This material exhibits high mechanical strength as a result of transformation toughening. During crack propagation, the stress generated at the crack tip induces a localized transformation of adjacent tetragonal grains into the monoclinic phase. This phase transformation is accompanied by volumetric expansion, which generates compressive stresses around the crack tip and thereby inhibits further crack propagation. The third generation of zirconia was developed by increasing the yttria concentration from 3 mol% to 5–6 mol%, resulting in improved translucency. These materials, commonly referred to as 5Y-zirconia or translucent zirconia, contain a higher proportion of the cubic phase, which enhances optical properties and esthetic performance compared with earlier zirconia generations. However, it was later recognized that the improved translucency of 5Y-zirconia was achieved at the expense of its mechanical strength. The fourth generation of zirconia, known as 4Y-zirconia, was developed to strike a balance between the high strength of traditional 3Y-zirconia and the improved translucency of 5Y-zirconia. With around 4 mol% yttria, it offers better optical properties while still retaining greater strength and fracture toughness than more translucent 5Y-zirconia. Finally, multi-yttria zirconia systems combining 3Y and 5Y, as well as 4Y and 5Y compositions, have been introduced to optimize the balance between mechanical performance and optical properties within a single restoration. This approach involves spatially varying the yttria content, with lower-yttria zirconia positioned in the cervical to middle third of the restoration to provide enhanced strength, and higher-yttria, more translucent zirconia used in the middle to occlusal regions to improve esthetics. This inverse relationship between translucency and fracture strength is a key consideration in implant prosthodontics [6,7,8]. Depending on its generation, zirconia can be used for the fabrication of inlays/onlays, veneers, single crowns, short- and long-span fixed partial dentures, implant abutments, resin-bonded anterior all-ceramic fixed partial dentures, and full-arch implant-supported rehabilitations [9,10,11,12,13].

High fracture strength is a critical requirement for implant-supported zirconia restorations due to the absence of the periodontal ligament and the reduced tactile sensitivity associated with dental implants. Occlusal loads on implant-supported restorations are often greater than those on tooth-supported restorations and are transmitted directly to the surrounding bone. Unlike natural teeth, implant-supported restorations are less capable of dissipating occlusal forces, making them more susceptible to mechanical complications such as fracture and chipping, particularly when brittle materials are used. Consequently, restorations with high fracture strength are essential to withstand repeated masticatory loading and to ensure long-term clinical durability and survival under intraoral conditions [14,15]. To meet these biomechanical demands, advanced design methodologies are required to optimize restoration geometry and improve stress distribution.

Generative design is a computer-assisted design approach in which a designer defines key parameters, such as material properties, functional requirements, anatomical limitations, and aesthetic considerations, while the software autonomously generates multiple optimized design solutions. In contrast to conventional CAD workflows, where the geometry is manually created by the operator, generative design algorithms evaluate numerous design possibilities and identify the most suitable configurations according to predefined criteria [16,17]. To achieve an optimal outcome, the acceptable range of parameter values must be carefully established before the design process begins, as these variables directly influence the mechanical and functional performance of the final configuration. Within implant prosthodontics, generative design offers the potential to evaluate a broad spectrum of structural configurations and identify the most favorable design for components such as hybrid implant abutments. However, the available literature provides limited data regarding standardized design parameters and biomechanical requirements. Recent studies dealing with generative design in implant prosthodontics investigated the transgingival and prosthodontic heights of implant abutments [18], as well as the emergence angle and emergence profile of implant-supported restorations [19]. Above-mentioned studies have highlighted the importance of the parametrization process prior to the application of generative design in hybrid implant abutment conceptualization. Accurate definition of design parameters is essential for achieving an optimal balance between mechanical resistance and peri-implant hard and soft tissue stability. Consequently, the integration of generative design principles into implant prosthodontics may contribute to the development of more individualized, biologically compatible, and mechanically efficient solutions. However, parameters influencing the fracture strength of implant-supported restorations have not yet been sufficiently addressed [2,4,9,14,15], despite their potential influence on the final design of hybrid implant abutments.

The aim of the present study was to systematically map and critically appraise the existing literature on the fracture strength of implant-supported zirconia restorations, with explicit attention to identify the parameters influencing their mechanical performance (e.g., fracture strength) and to determine the optimal values of these parameters. To identify current gaps in the evidence, a scoping review was conducted to identify the essential preparatory steps required for the successful implementation of generative design in implant prosthodontics.

## 2. Materials and Methods

The present study was prospectively registered in the Open Science Framework (OSF) public registry (Registration DOI: 10.17605/OSF.IO/8SG25) and conducted in accordance with the PRISMA Extension for Scoping Reviews (PRISMA-ScR) guidelines [20]. A scoping review methodology was selected to systematically map and synthesize the available evidence concerning fracture strength of implant-supported zirconia restorations. Eligibility criteria for this scoping review were established in accordance with the Population–Concept–Context (PCC) framework, as recommended by the Joanna Briggs Institute (JBI) for the conduct of scoping reviews.

P: Implant-supported zirconia restorations across a range of clinical configurations.

C: The core concept under investigation was the range of parameters influencing the fracture strength of implant-supported zirconia restorations.

C: The contextual boundary of this review was restricted to in vitro studies, given that such experimental designs allow for standardized, controlled evaluation of the biomechanical behavior of implant-supported zirconia restorations under simulated functional conditions.

**Figure 1:**
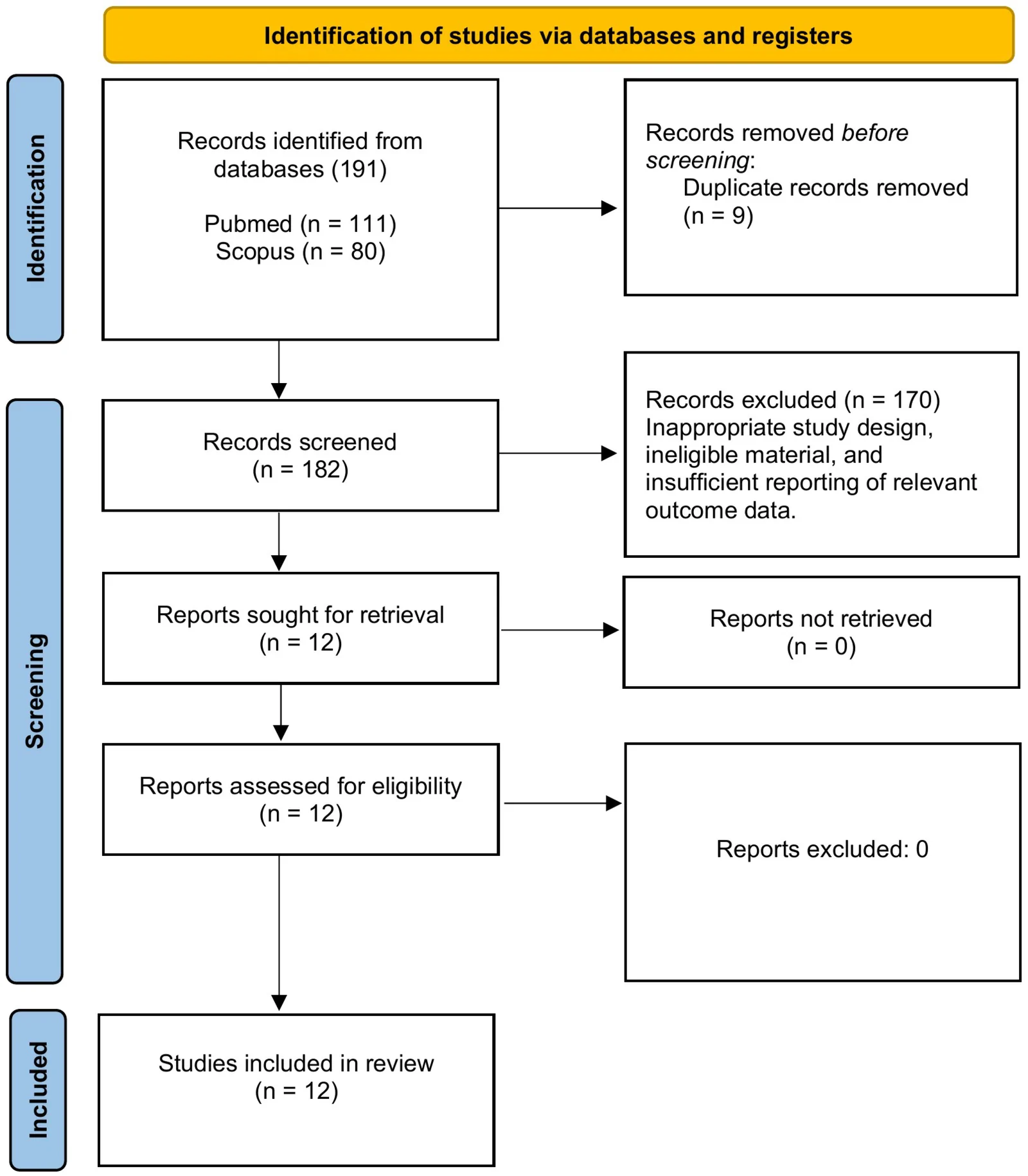
PRISMA-based flowchart illustrating the study selection process.

In vitro studies published in English within the past ten years that reported parameters influencing the fracture strength of implant-supported zirconia restorations were considered eligible to capture recent developments and ensure the inclusion of up-to-date and clinically relevant evidence. Exclusion criteria comprised case series, editorials, and interview publications, as these designs do not provide sufficiently robust or generalizable data for the purposes of this review. A comprehensive literature search was conducted in the PubMed and Scopus databases between 1 December 2025 and 1 May 2026, to identify relevant studies published by the end of the search period. The search strategy was structured to address the outcome of interest. An electronic search of the selected databases was conducted using the terms “fracture strength” and “implant-supported zirconia restoration,” combined with the Boolean operator AND. The detailed PRISMA checklist is provided in the Supplementary Materials [20]. The study selection process followed a structured, multi-stage approach. Initially, two experienced reviewers (S.G. and V.P.) independently screened the titles of all retrieved records against the predefined inclusion criteria. Subsequently, abstracts of potentially relevant studies were evaluated, followed by full-text assessments of studies meeting the eligibility requirements. During the full-text assessment, studies were excluded because of an inappropriate study design, an ineligible material, or insufficient reporting of relevant outcome data. Final inclusion was determined through consensus between the primary reviewers. In cases of disagreement or uncertainty, an additional reviewer (A.C.) was consulted to achieve resolution, ensuring methodological rigor and minimizing selection bias. Data extraction was performed systematically and included the following variables: authors, year of publication, title of study, type of study, sample size, and reported outcomes. The extracted data are summarized in Table 1. The study selection process is illustrated using a PRISMA flow diagram (Figure 1), which presents the final number of studies included in the review.

## 3. Results

Initially, a total of 191 studies were identified through searches of the PubMed and Scopus databases. Following the removal of nine duplicate records, 182 studies proceeded to the screening stage. Finally, 12 studies fulfilled the inclusion criteria and were included in the final analysis. Figure 1 presents a PRISMA (Preferred Reporting Items for Systematic Reviews and Meta-Analyses) flowchart illustrating the process of identification, screening, eligibility assessment, and final selection of the studies retrieved during the literature search. The fracture strength of implant-supported zirconia restorations was investigated in twelve studies [21,22,23,24,25,26,27,28,29,30,31,32], and the findings are summarized below.

### 3.1. Restorations Design

Takano et al. [21] investigated fracture strength of hybrid abutment crowns. Authors reported that monolithic translucent zirconia restorations showed the higher median fracture strengths (2.06 kN), significantly outperforming bi-layered zirconia restorations (1.12 kN). However, both restorations can withstand the maximum occlusal forces expected in the premolar region. Moreover, Nazari et al. [22] investigated the fracture strength of implant-supported fixed partial dentures with excessive crown height. For zirconia restorations, the average load was 2086 ± 362 N, suggesting that zirconia may be capable of withstanding the occlusal forces typically encountered in the molar region, even in cases of excessive crown height [22].

### 3.2. Abutment Type

Kim et al. [23] reported a fracture strength of 548.03 ± 77.87 N for multilayered zirconia crowns on titanium abutments.

### 3.3. Surface Modifications

Park et al. [24] found that unmodified zirconia-titanium base restorations had significantly higher fracture strength (6154.84 ± 320.50 N) than modified and polished restorations (5593.13 ± 486.51 N) suggesting that reducing cervical zirconia thickness may weaken the restoration even though the values remained above human chewing forces.

### 3.4. Material Thickness

Kang et al. [25] examined fracture strength of three various thicknesses (1.0 mm, 1.5 mm, and 2.0 mm) of multilayer monolithic zirconia restorations cemented on metal abutments. Multilayer monolithic zirconia crowns maintained fracture strengths above 1200 N at all tested thicknesses, even at a thickness of 1.0 mm. The results indicate that thickness influenced fracture strength; however, all measured values exceeded typical occlusal forces.

### 3.5. Retention Type

Affendi et al. [26] reported that monolithic cement-retained zirconia crowns achieved the highest fracture strength (2955.84 ± 310.93 N), followed by monolithic screw-retained crowns (2846.11 ± 370.59 N), whereas bilayer system crowns showed significantly lower values (1994.70 ± 95.44 N). In addition, Çötert et al. [27] reported that hybrid-abutment crowns exhibited higher fracture strength values than screwmentable crowns, with titanium–porcelain abutment crowns demonstrating the highest fracture strength (385.84 ± 27.68 N), followed by zirconia–porcelain abutment crowns (313.18 ± 39.97 N), titanium screwmentable crowns (272.69 ± 35.03 N), and zirconia screwmentable crowns (156.71 ± 19.83 N). Obermeier et al. [28] reported fracture loads of 853.7 ± 376.6 N for the screw-retained zirconia group and 729.7 ± 475.0 N for the cement-retained zirconia group. Results indicate that type of retention did not have influence on fracture loads. Yazigi et al. [29] evaluated the fracture strength of one-piece screw-retained implant-supported hybrid abutment crowns. The zirconia group exhibited a median fracture strength of 2645 N.

### 3.6. Material Selection

Arısan et al. [30] investigated the effect of various abutment–crown material combinations on the fracture strength of implant-supported restorations. The results demonstrated that zirconia abutment–zirconia crown restorations exhibited the highest mean fracture strength (1417 N), indicating their suitability for posterior restorations.

### 3.7. Luting Agents

Moriya et al. [31] evaluated the impact of various luting agents on the fracture load of implant-supported prostheses. The median fracture load values were the highest for monolithic yttria-partially stabilized zirconia restorations (3.89 kN, 3.42 kN, and 3.16 kN), independent of the luting agent.

### 3.8. Interface Characteristics

Rutkūnas et al. [32] identified horizontal misfit as a major contributor to reduced fracture strength, reporting fracture loads of 1509.20 ± 179 N and 1557.25 ± 283.67 N.

Above-mentioned studies [21,22,23,24,25,26,27,28,29,30,31,32] investigating the fracture strength of implant-supported zirconia restorations are presented in Table 1.

## 4. Discussion

Implant therapy using two-piece implant systems involves a multistage treatment process comprising both surgical and prosthodontic components. The surgical component consists of a dental implant, typically fabricated from titanium because of its favorable biocompatibility, which is surgically inserted into the alveolar bone of the maxilla or mandible. The implant abutment serves as a critical connecting element between the implant fixture and the definitive prosthodontic restoration. Appropriate abutment selection is therefore essential for achieving optimal functional and esthetic outcomes and ensuring the long-term success of implant-supported restorations [18,33,34].

Stock implant abutments offer several advantages, including cost-effectiveness, reliable engagement with the implant body, and the possibility of intraoral or extraoral modification. However, their use is associated with certain limitations, including prolonged preparation time, particularly in cases involving improperly positioned implants. Furthermore, because prefabricated abutments may not adequately conform to the anatomical complexity of the gingival contours, achieving an optimal emergence profile and esthetic outcome can be challenging. Titanium abutments are widely used because of their well-documented biological and mechanical properties. However, their grayish appearance may become visible through the peri-implant mucosa, particularly in patients with a thin mucosal phenotype, thereby compromising the optical and esthetic outcomes of implant-supported restorations. Zirconia abutments offer more favorable optical properties; nevertheless, their clinical application is associated with several limitations, including the risk of abutment fracture and wear of the titanium implant at the implant–abutment interface [18,35].

Advances in Computer-Aided Design/Computer-Aided Manufacturing (CAD/CAM) technology have facilitated the increasing use of digital workflows for implant abutment fabrication. This approach reduces the number of manual procedures, simplifies the manufacturing process, and provides a high degree of predictability. CAD/CAM-fabricated abutments can also be customized according to the anatomical and clinical requirements of each patient and manufactured from various materials, most commonly titanium and zirconia. The hybrid-abutment concept represents a contemporary approach aimed at achieving favorable esthetic and mechanical outcomes. Hybrid implant abutments combine the esthetic and biological advantages of zirconia with the mechanical reliability of titanium while preserving the integrity of the implant–abutment interface [18,36,37,38].

Prior to the implementation of generative design in implant prosthodontics, a detailed parametrization procedure must be established to define the biomechanical variables influencing the design process. This methodology allows the generative workflow to remain constrained within clinically acceptable limits, ensuring that the resulting designs are both anatomically precise and biomechanically functional under intraoral conditions. Parametrization represents a critical basis of the design strategy, as it enables the system to identify and evaluate the parameters most relevant to each specific clinical situation. In the absence of appropriate parametrization, the generated prosthodontic solutions may not adequately fulfill the functional and clinical demands associated with implant-supported restorations. Accordingly, the present study was designed to analyze and synthesize the available evidence on the parameters influencing the fracture strength of implant-supported zirconia restorations.

Twelve studies [21,22,23,24,25,26,27,28,29,30,31,32] (Table 1) examined the fracture strength of implant-supported zirconia restorations and reported the following findings. Takano et al. [21] evaluated the fracture strength of hybrid abutment crowns manufactured using different CAD/CAM restorative materials. Two types of zirconia restorations were evaluated, including bi-layered zirconia restorations and translucent zirconia (4Y-PSZ) restorations, both of which were adhesively bonded to titanium abutments. The results indicated that monolithic 4Y-PSZ zirconia restorations used for hybrid abutment crowns demonstrated superior fracture strength when compared with bi-layered 3Y-TZP restorations in the premolar region. These findings may be attributed to differences in the physical and mechanical properties of the evaluated restorative materials (e.g., flexural strength). In the bi-layered zirconia group, fracture of the veneering porcelain was observed in all specimens, whereas the underlying 3Y-TZP frameworks remained intact. Veneering porcelain fracture is a common technical complication associated with implant-supported restorations. However, the minimum fracture strength recorded for all tested specimens exceeded 0.7 kN, surpassing the maximum reported premolar masticatory force of approximately 0.47 kN [21]. Nazari et al. [22] investigated how the choice of restorative material affected the fracture strength of implant-supported fixed partial dentures with excessive crown height (15 mm). Three-unit frameworks featuring a supportive anatomical design were fabricated from different materials, including zirconia. The zirconia group exhibited a load of 2086 ± 362 N indicating that zirconia possesses sufficient mechanical strength to withstand physiological occlusal forces in the molar region [22]. Furthermore, Kim et al. [23] compared fracture strength of multi-layered zirconia implant-supported crowns on titanium implant abutment with fracture strength of lithium disilicate crowns on titanium and zirconia implant abutments. The fracture strength of the implant-supported crown was significantly greater in the titanium abutment group (548.03 ± 77.87 N) compared with the zirconia abutment group (501.52 ± 30.69 N; 283.58 ± 43.70 N). Crown fracture was observed in the zirconia abutment–lithium disilicate crown group. In contrast, the groups restored with lithium disilicate and zirconia crowns supported by titanium abutments demonstrated only the presence of crack lines within the crown structure, without evidence of complete fracture [23]. Park et al. [24] evaluated whether modification of the submucosal cervical contour in implant-supported zirconia–titanium base (Zi–Ti base) restorations resulted in a significant difference in fracture strength compared with Zi–Ti base restorations without modification of the cervical submucosal region. Fracture strength values ranged from 4354.68 to 6412.49 N in the modified test group and from 5400.31 to 6953.22 N in the control group. The control group demonstrated a significantly higher mean fracture strength (6154.84 ± 320.50 N) compared with the modified group (5593.13 ± 486.51 N). These findings indicate that modification of the submucosal contour adversely affected the fracture strength of the restorations. Although zirconia is well known for its high mechanical strength, results suggest that cross-sectional thickness may also contribute to fracture strength. Nevertheless, the mean fracture strength values recorded in both groups remained higher than the maximum physiological masticatory forces observed in humans [24]. Kang et al. [25] aimed to determine the minimum thickness required for monolithic zirconia crowns. Three various monolithic zirconia thicknesses (1.0 mm, 1.5 mm, and 2.0 mm) and three tooth positions (incisor, premolar, and molar) were selected and bonded to the 2.0 mm metal abutments. Monolithic zirconia crowns exhibited a significant increase in strength as the crown thickness increased across all tooth positions. Nevertheless, all crowns demonstrated sufficient mechanical strength at a thickness of 1.0 mm. Despite the reduction in thickness, the monolithic zirconia crowns demonstrated a remarkable capacity to maintain fracture strength values exceeding 1200 N [25]. Affendi et al. [26] investigated the fracture strength of implant crowns fabricated using monolithic cement-retained (MCV), monolithic screw-retained (MSV), and bilayer designs (BSV), all of which were bonded to titanium-base abutments. The MCV crowns exhibited the highest fracture strength (2955.84 ± 310.93 N), followed by the MSV crowns (2846.11 ± 370.59 N). The BSV group showed the lowest fracture strength (1994.70 ± 95.44 N). The higher fracture strength observed in the MCV crowns may be related to their monolithic design, which preserves structural continuity by eliminating internal interfaces and screw-access openings. The absence of screw-access holes may reduce stress concentration within the restoration, thereby improving its mechanical stability under functional loading compared with screw-retained designs. The presence of screw-access holes has the potential to compromise the structural integrity of the ceramic. Consequently, the crown design influences the fracture load of implant-supported crowns [26]. Çötert et al. [27] investigated the fracture strength of hybrid-abutment and screwmentable crowns, including SM-Ti (screwmentable titanium–porcelain crowns on stock titanium abutments), SM-Zr (screwmentable zirconia–porcelain crowns on stock zirconia abutments), AC-Ti (titanium–porcelain abutment crowns), and AC-Zr (zirconia–porcelain abutment crowns). The titanium–porcelain group exhibited the highest fracture strength (385.84 ± 27.68 N), followed by the zirconia–porcelain group (313.18 ± 39.97 N), screwmentable titanium–porcelain group (272.69 ± 35.03 N), and screwmentable zirconia–porcelain group (156.71 ± 19.83 N). The evaluation of fracture strength revealed that hybrid-abutment crowns demonstrated greater mechanical strength and durability than screwmentable crown designs. Fabrication of the system as a single-piece structure, without the presence of an intermediate cement layer, enhances the overall mechanical integrity of the prosthodontic restoration. Consequently, the use of hybrid-abutment crowns may provide clinical advantages by improving the resistance of implant-supported restorations to excessive occlusal loading and masticatory forces [27]. Additionally, Obermeier et al. [28] compared the fracture strength of implant-supported all-ceramic single crowns manufactured using different fabrication and fixation concepts. Several groups of CAD/CAM-fabricated all-ceramic single crowns were investigated: CZL (cement-retained layered zirconia), SZL (screw-retained layered zirconia), MZL (screw-retained layered zirconia with a modified coping design), and SST (screw-retained zirconia crowns veneered with sintered lithium disilicate). No statistically significant differences in mean fracture load were observed among the tested groups. Fracture loads of 853.7 ± 376.6 N, 931.7 ± 235.2 N, and 729.7 ± 475.0 N were recorded for the SZL, MZL, and CZL groups, respectively. The type of restoration retention (screw-retained vs. cemented) and the type of ceramic material did not significantly affect the load at which the restoration fractured [28]. Yazigi et al. [29] evaluated the fracture strength of commonly used ceramic materials for one-piece screw-retained implant-supported hybrid abutment crowns. 3Y-TZP zirconia was one of the restorative materials evaluated in the study. The highest median fracture strength was observed in the zirconia group, with a value of 2645 N. The choice of restorative material affected the fracture strength of screw-retained hybrid abutment crowns, with zirconia exhibiting the highest values [29]. Arısan et al. [30] investigated the fracture strength of implant-supported restorations fabricated using different implant abutment–crown material combinations. Abutments were fabricated from three monolithic materials: 5Y-tetragonal zirconia polycrystal (5Y-TZP), lithium disilicate, and a ceramic-reinforced polymer. Similarly, crowns were manufactured from three monolithic CAD/CAM materials: 5Y-TZP zirconia, advanced lithium disilicate, and a hybrid nanoceramic. All groups demonstrated mean fracture loads greater than 500 N. However, the highest mean strength was observed in the zirconia–zirconia group (1417 N), followed by lithium-disilicate–zirconia group (1349 N). The superior strength demonstrated by zirconia-based restorations suggests their potential suitability for posterior regions characterized by high functional demands [30]. Moreover, Moriya et al. [31] evaluated several restorative materials including monolithic yttria-partially stabilized zirconia (MPZ) restorations and porcelain-layered yttria-stabilized tetragonal zirconia polycrystal (PLZ) restorations. The specimens were luted using either an adhesive resin luting agent (RLA), glass ionomer cement (GIC), or zinc phosphate cement (ZPC). The fracture loads of the monolithic yttria-partially stabilized zirconia specimens were significantly higher than those of all other test groups, irrespective of the luting agent used. The present findings indicate that cementation with an adhesive resin luting agent (RLA) increased the fracture loads of implant-supported monolithic Y-PSZ (MPZ) restorations in the premolar area [31]. Rutkūnas et al. [32] evaluated the effects of fatigue and prosthetic misfit on the load-bearing capacity of cement-retained and screw-retained two-implant-supported fixed dental prostheses. The authors reported a significant difference between the horizontal misfit group and the control group, indicating that horizontal misfit could reduce load-bearing capacity [32].

Optimal mechanical properties (e.g., fracture strength) of implant-supported zirconia restorations are essential to enhance long-term clinical performance. With the application of digital technologies and generative design it is possible to design structures with precise dimensions. Several parameters, including the transmucosal and prosthodontic height of the implant abutment, as well as the emergence angle and emergence profile of implant-supported restorations, have already been analyzed, and their influence on peri-implant hard and soft tissues has been reported [18,19]. The outcomes of the present study will be applied to improve the parametric model which will be used in the manufacturing process of hybrid implant abutment.

Three limitations should be acknowledged: the small number of studies available for analysis, the restriction of the literature search to two databases, and the substantial heterogeneity among the included studies.

## 5. Conclusions

Within the limitations of the present study, it can be concluded that the implant-supported zirconia restorations included in this review generally exhibited fracture strength values exceeding physiological masticatory forces. Overall, restoration design, abutment type, surface modification, material thickness, retention mode, material selection, luting agent, and interface characteristics appear to be important factors influencing the fracture strength of implant-supported zirconia restorations. Above-mentioned parameters will be taken into consideration during the development of the parametric model and their implementation within the generative design workflow for implant prosthodontic applications (e.g., hybrid implant abutment).

## 6. Future Directions

Long-term clinical investigations are needed to validate in vitro findings and assess the influence of functional loading, aging, and oral environmental factors on implant-supported zirconia restoration performance. Future studies should evaluate the combined effects of restoration design, zirconia generation, cementation strategy, and implant-abutment configuration on fracture strength values. Furthermore, the integration of these biomechanical findings into advanced parametric models for generative design may facilitate the development and optimization of hybrid implant abutments, enabling the creation of patient-specific solutions with enhanced mechanical performance and long-term clinical reliability.

## Figures and Tables

**Table 1:** Studies reporting fracture strength of implant-supported zirconia restorations.

Authors, Year	Title	Study Type	Sample Size	Outcome
Takano et al. (2023) [21]	Fracture strength of implant-supported hybrid abutment crowns in premolar region fabricated using different restorative CAD/CAM materials	In vitro study	44 specimens	Monolithic translucent zirconia restorations exhibited the highest fracture strength, significantly outperforming bi-layered zirconia restorations
Nazari et al. (2016) [22]	Fracture strength of three-unit implant supported fixed partial dentures with excessive crown height fabricated from different materials	In vitro study	30 specimens	Implant supported three-unit fixed partial dentures fabricated from zirconia in excessive crown height space have the potential to withstand occlusal forces
Kim et al. (2025) [23]	Impact of liner treatment on the translucency of CAD/CAM multi-colored lithium disilicate and multi-layered zirconia implant-supported crowns, and evaluation of fracture strength of ceramic crowns	In vitro study	36 specimens	Fracture strength was highest in titanium abutments and lowest in zirconia abutment
Park et al. (2022) [24]	Evaluation of fracture resistance of zirconia modification/polishing around implant abutments	In vitro study	40 specimens	Modification of submucosal contour significantly decreased the fracture strength
Kang et al. (2024) [25]	Fracture characteristics and translucency of multilayer monolithic zirconia crowns of various thicknesses	In vitro study	135 specimens	For multilayer monolithic zirconia crowns, optimal mechanical integrity can be maintained at a minimum thickness of 1.0 mm
Affendi et al. (2025) [26]	The effect of crown design on fracture strength and mode of implant-supported molar crown bonded to titanium-based abutment	In vitro study	30 specimens	Monolithic cement-retained crowns demonstrated superior mechanical strength
Çötert et al. (2025) [27]	In vitro comparison of screw loosening, fracture strength and failure mode of implant-supported hybrid-abutment crowns and screwmentable crowns manufactured with different materials	In vitro study	40 specimens	Hybrid-abutment crowns have higher fracture strength than screwmentable crowns
Obermeier et al. (2018) [28]	Mechanical performance of cement- and screw-retained All-ceramic single crowns on dental implants	In vitro study	50 specimens	Mode of retention (screw-retained or cemented) did not influence on the fracture load
Yazigi et al. (2020) [29]	The influence of the restorative material on the mechanical behavior of screw-retained hybrid-abutment-crowns	In vitro study	40 specimens	The fracture strength of screw-retained hybrid abutment crowns is influenced by the restorative material, with zirconia demonstrating the highest fracture strength
Arısan et al. (2025) [30]	Fracture strength of implant-supported hybrid abutment crowns: an in vitro study of ceramic and polymer-based materials in the premolar region	In vitro study	90 specimens	Zirconia-based restorations demonstrated the highest resistance to fracture
Moriya et al. (2020) [31]	Effect of luting agent type on fracture loads of implant-supported ceramic premolar prostheses	In vitro study	99 specimens	Fracture loads were significantly higher for monolithic yttria-partially stabilized zirconia restorations specimens, regardless of luting agent
Rutkūnas et al. (2025) [32]	Influence of misfit direction and abutment type on torque loss and fracture strength of implant-supported fixed dental prostheses	In vitro study	80 specimens	Horizontal misfit was identified as the critical factor reducing fracture strength

## Data Availability

No new data were created or analyzed in this study. Data sharing is not applicable to this article.

## Supplementary Materials

The following supporting information can be downloaded at: https://www.mdpi.com/article/10.3390/jfb17080391/s1, Table S1: Preferred Reporting Items for Systematic reviews and Meta-Analyses extension for Scoping Reviews (PRISMA-ScR) Checklist.
